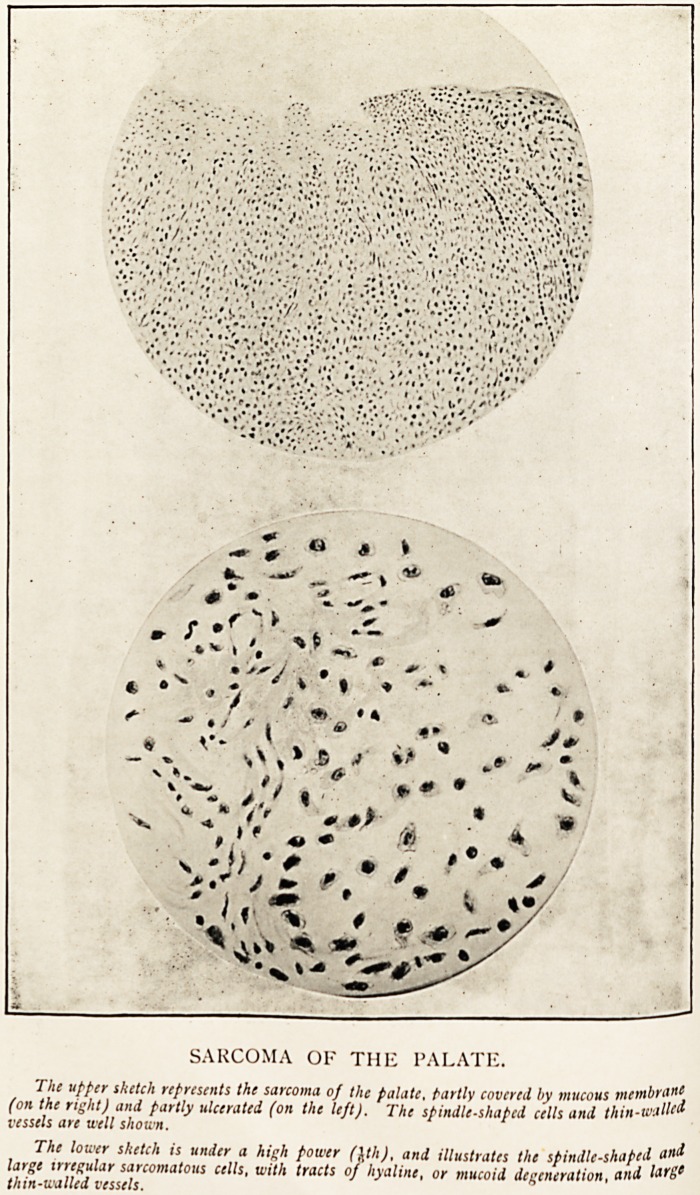# Spindle-Celled Sarcoma of the Hard Palate

**Published:** 1900-09

**Authors:** W. J. Greer


SARCOMA OF THE PALATE.

*The upper sketch represents the sarcoma of the palate, partly covered by mucous membrane (on the right) and partly ulcerated (on the left). The spindle-shaped cells and thin-walled vessels are well shown.*

*The lower sketch is under a high power ($\frac{1}{8}$th), and illustrates the spindle-shaped and large irregular sarcomatous cells, with tracts of hyaline, or mucoid degeneration, and large thin-walled vessels.*

advise passing the cord through a sort of button-hole made in the os pubis. Such procedures are more the result of inordinate mechanical originality than the product of a wise application of the lessons which Nature affords. The surgeon may take it for granted that the mechanical devices wrought by evolution are sounder than the machinations of his own imagination.

---

### SPINDLE-CELLED SARCOMA OF THE HARD PALATE.

BY

W. J. Greer, F.R.C.S. Irel.

---

The literature on this subject is not by any means voluminous, the reason being, no doubt, the rarity of the condition; yet the field of medical print, though not abundantly fertile, has produced a harvest which is already reaped through the painstaking labours of Mr. Paget[1]; to the dozen cases, I venture to add another, and at the same time to inflict a dissertation on a long-suffering medical public.

The patient was a young man, aged 26 years, who enjoyed fair health (save for an accident which some years ago necessitated amputation through the right shoulder-joint) until his present trouble; his family history furnishes a paternal grandfather who suffered from "cancer of the lip," and his father at the moment is suffering from multiple nodular growths of the peritoneal cavity, possibly carcinoma. The patient presented himself to his medical man early in December, 1899, for treatment of a small sore on the roof of his mouth; this gradually increased in size, and the pain, which was always considerable, became agonizing. Treatment by cauterization was carried out without any benefit. His doctor recognized the seriousness of the condition, and gave a grave prognosis. In January of this year he came under my care. His general health was excellent, and, but for the pain, he himself would have paid little attention to the mouth: on examining the latter one could see on the right side of the hard palate, opposite the first molar, but close to the median raphé, an ulcer about the size of sixpence; the base was inflamed and raised, the floor covered with small grey granulations, with just a little pus; the edges hard, sinuous,

[1] "Tumours of the Palate," *St. Bart.'s Reports*, 1886.

and everted; the whole appearance carrying the conviction of epithelioma. No account of an injury could be elicited, nor was there a history or signs of syphilis; no enlarged gland to be detected.

I felt obliged to confirm the opinion, already expressed, that the disease was highly dangerous, and to complete the diagnosis I removed a small portion for the microscope. This was forwarded to the Clinical Research Association, and a report asked for by telegram; the reply came that the specimen was "not malignant"; no doubt, but that the clinical history being so apparently that of an epithelioma, this morbid condition only was immediately sought for. In the face of this report, and the pain being so severe, I determined to freely remove the ulcer. This I did under chloroform; the operation gave the patient almost immediate relief from the pain, and it has never been so intense since. A day or two following the telegram, Mr. Targett very kindly wrote me:—"The specimen from the hard palate has not given a sufficiently definite result for an opinion as regards malignant disease, therefore it will be better to wait until a second series of sections have been prepared. The general features are those of chronic inflammation —not growth—but I have met with a dense form of epithelioma in the palate which was not unlike this."

The patient was now progressing favourably, and I was almost inclined to be hopeful; but on January 23rd, only six days from the receipt of the specimen, I received the final report from Mr. Targett. It has proved, alas! as accurate as it is brilliant. He says: "Further examination of this specimen confirms the previous report—that it is not an epithelioma. Owing to the ulceration there is a thick layer of inflammatory and necrotic tissue in the base of the ulcer. But beneath this there lies a thin stratum of spindle-celled tissue which I think must be regarded as sarcomatous. After a most careful examination I feel convinced that the new formation is really a spindle-celled sarcoma, and that this has become covered with much inflammatory tissue in consequence of the ulceration."

After this report I felt that the only chance the patient had was in removal of both superior maxillæ. This or any further operation was declined. The disease has followed the course of first completely involving and destroying the hard and soft palates, then invading the right maxillary antrum and expanding, the growth reaching the zygomatic fossa; meanwhile the pressure below and to the outside had entirely crushed the right eye, the apparent route to the cranial cavity being through the spheno-maxillary fissure, then the sphenoidal fissure and optic foramen; the basi-sphenoid became thickly infiltrated through direct extension from the palate. The whole right side of the face was distorted into a large tumour, which had ulcerated in several places, and discharged exceedingly foul sanious pus. The poor fellow, exhausted by pain, hemorrhage, and septic absorption, died on June 29th.

It would appear to be almost fruitless, with our present pathological knowledge, to discuss the cause of such a condition. One can talk of fœtal remnants, and insinuate a bacteriological stimulus, and this seems to be about as far as we can go; indeed, the inclusion of fœtal tissue is readily suggested by the "fact that nowhere more than in the palate does the body suffer from arrest and perversion of development."[1]

Professor Ziegler[2] states that "Sarcomata originate in structures belonging to the connective tissue group, *i.e.*, in formed or unformed fibrous tissue, in cartilaginous, bony, mucous, lymphoid, neurogliar, or adipose tissue. The transformation of these into tumour tissue is effected by the growth and multiplication of the constituent cells. It is of great interest to note that cells which form part of what we might call congenital heteroplastic foci may often serve as the starting-point of sarcoma."

Here then in the palate we may have all the essential elements lying dormant; but what stimulates them into proliferation? Is it a bacillus?

Mr. Paget's researches show that sarcoma of the palate occurs equally in man and woman, more in the soft than in the hard palate, more common on left side than on right, and the average age would be about forty. They are sometimes encapsuled, though this was not so in my case, as witness

[1] "Tumours of the Palate," *St. Bart.'s Reports*, 1886.

[2] *General Pathological Anatomy*, 1897.

Mr. Christopher Heath's[1] case, in which the growth "shelled out with a distinct capsule." Mr. Heath also saw Sir William Fergusson remove a similar growth half-an-inch in diameter.

Evidently the tumours seem mostly to be of the round-celled variety, Mr. Treves's[2] case, No. 11 in Mr. Paget's list, being the only one in the list distinctly stated to be spindle-celled.

It is, perhaps, profitable to remark in conclusion how rare these growths of the palatine process of the jaw are, in comparison with the sarcomatous epulides of the alveolar process, and also the much greater infrequency of their occurrence in the hard than in the soft palate.

Finally I must express my sincere thanks to the Clinical Research Association for the indispensable help afforded me, and also for the very beautiful drawing, reproduced with this article.

[1] *Injuries and Diseases of the Jaw*, 1894.

[2] *Lancet*, 1886, ii. 869.

## Figures and Tables

**Figure f1:**